# Colonization of Warsaw by the red fox *Vulpes vulpes* in the years 1976–2019

**DOI:** 10.1038/s41598-021-92844-2

**Published:** 2021-07-06

**Authors:** Mateusz Jackowiak, Jakub Gryz, Karolina Jasińska, Michał Brach, Leszek Bolibok, Piotr Kowal, Dagny Krauze-Gryz

**Affiliations:** 1 Central Laboratory for Environmental Analysis - CentLab, Institute of Environmental Protection - National Research Institute, Krucza 5/11D, 00-548 Warsaw, Poland; 2 Department of Forest Zoology and Wildlife Management, Institute of Forest Sciences, Warsaw University of Life Sciences WULS-SGGW, Nowoursynowska 159, 02-776 Warsaw, Poland; 3 Department of Forest Ecology, Forest Research Institute, Braci Leśnej 3, Sękocin Stary, 05-090 Raszyn, Poland; 4 Department of Geomatics and Land Management, Institute of Forest Sciences, Warsaw University of Life Sciences WULS-SGGW, Nowoursynowska 159, 02-776 Warsaw, Poland; 5 Department of Forest Silviculture, Institute of Forest Sciences, Warsaw University of Life Sciences WULS-SGGW, Nowoursynowska 159, 02-776 Warsaw, Poland

**Keywords:** Ecology, Zoology, Ecology

## Abstract

The red fox is one of the most adaptable carnivores inhabiting cities. The aim of our study was to describe the process of Warsaw colonization by the red fox. We focused on: (1) the fox distribution in Warsaw on the basis of presence-absence data (2005–2012) over a grid of 1 × 1 km^2^, (2) the process of settlement in 29 green areas (study periods 1976–1978, 2004–2012, and 2016–2019) in relation to habitat type, and (3) temporal and spatial patterns of the red fox incidents (1998–2015) reported by Warsaw citizens. We found out that: (1) the red fox penetrated the whole city (i.e. its presence was confirmed in all squares of the grid), (2) 21% of the green areas were colonized in 1976–1978 but 93% in 2016–2019. Forests and riparian habitats were occupied more frequently than parks and cemeteries in 1976–1978 with no difference in the further years; (3) the probability of the fox incidents increased over years, was higher in June-October, on working days, and around noon, and with the share of discontinuous urban fabric in the buffers around incident locations. Nevertheless, the incidents only partially reflect population abundance trends and activity patterns of the species, so should be treated cautiously.

## Introduction

For decades, the use of urban areas by wildlife and urban species population numbers have increased^1–6^, which is a result of wildlife adaptation to specific urban conditions (like high human density, large areas of impervious surfaces and built-up areas)^7–10^. One of species inhabiting urban areas is the red fox (*Vulpes vulpes*)^11^. Thanks to its plasticity the red fox utilizes a wide range of habitats—from near-natural to built-up areas^12–15^, and uses a variety of food in different habitats^16–19^. As a result, urban populations of the red fox may reach extremely high densities^1,13,20–23^. This in turn may lead to emerging and rapidly spreading epizooties (e.g. sarcoptic mange), limiting the number of foxes^24,25^. The occurrence of the red fox in urban areas, may result in predation on pets and digging burrows^16,26–29^, which is annoying and frequently reported by city inhabitants^30,31^.

The first red foxes in urban areas were recorded in the 1930s in the suburbs of London^12,32^. Yet, historical analyses showed that foxes used urban habitats in the mid-nineteenth century^33^ or even earlier^34^. The time of colonization of urban areas by red fox varies by countries: Denmark—the 1960s^33^, Australia—the 1970s^35^, Switzerland—the 1980s^36^. Nowadays, red fox populations have been recorded from more than one hundred cities around the world, mostly in Great Britain and other European countries^37^, but new cities have been colonized^28^ and some urban populations have continued to increase in numbers^31^. The red fox populations expanding in urban areas are probably related to anti-rabies vaccinations implemented in many countries^36,38^, but not all. Although the red fox is common in many cities, breeding in typically urban populations have not been recorded from everywhere^34^.

The red fox is mainly nocturnal^31,39^. Moreover, patterns of daylight encounters of the red fox are variable^21^ cf.^40^, and the probability of fox sighting are influenced by human activity and their space use in a city^31^. Therefore, the increase of the red fox sightings does not necessarily reflect increase in the population abundance, but can be related to the rising number of the red fox-human conflicts (traffic collisions or intervention of relevant services, e.g. trapping, interventional culling)^28,41^. Taking all this into account, direct observations can be used to estimate abundance of urban populations of red foxes^20,42^, yet estimation bias needs to be acknowledged^43^. On the other hand, registry of incidents involving foxes (e.g. traffic collisions), was found to be quite a reliable indicator of population rise, changes in species distribution^5,21,41^ and a possible density estimator^44^.

Preliminary studies on red fox population in Warsaw, conducted in the 1970s, showed a very low population density and just incidental (and associated to forest areas in peripheral city zones) records of breeding [Goszczyński J., unpublished]. It is likely that the population has grown since then and has spread throughout the city area. Therefore, the aim of our study was to describe the process of Warsaw colonization by the red fox on the basis of current and historical field work and red fox incident reports. The study included three parts. First, we checked what proportion of the city was colonized by the red fox on the basis of presence–absence data over a grid of squares. We assumed that red foxes would be present mostly in outer city districts, forests and continuous fields and meadows, but also within the Vistula river riparian forests, as this would serve as an ecological corridor. Next, we analysed the progress in the settlement of selected urban green areas by red fox for which historical and present field work data were available. We related the process of colonization to habitat types of those areas. Additionally, we analysed how the number of red fox incidents changed over a twenty year period as this would, at least to some extent, reflect population abundance increase. We also checked what were the temporal and spatial factors shaping the pattern of red fox incidents and related this to both carnivore biology and ecology but also human activity within the city. This would help to evaluate usefulness of such data to document population increase of the red fox in urban conditions.

## Methods

### Study area

The research was conducted in Warsaw (52° 13′ 47″ N 21° 00′ 42″ E), the capital city of Poland, the largest (517 km^2^) and the most populous (1,778,000 inhabitants, 3437 inhabitants/km^2^) city of the country^45^. The city is situated at an altitude of around 113 m above sea level with an annual rainfall of about 500 mm and an average annual temperature of 7.7 °C^46^.

**Figure 1:**
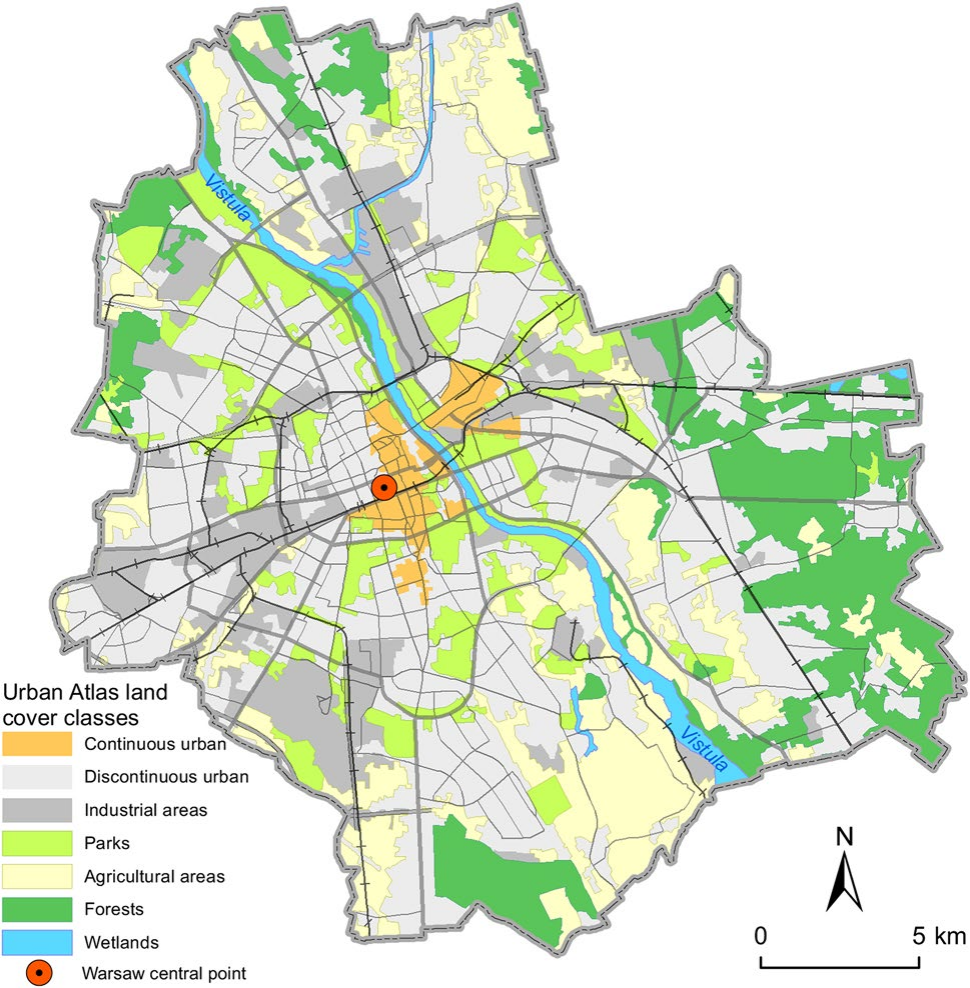
Distribution of green areas and other habitats in Warsaw. The Palace of Culture and Science, assumed as the most central point of the city is marked.

The Vistula River flows through Warsaw, dividing the city into two parts (Fig. 1). The left bank of the river is characterized by a high degree of anthropogenic transformation, while along the right bank natural riparian forests, included in the Natura 2000 network^47–49^ and constituting an important ecological corridor, have been preserved.

Warsaw is divided into 18 districts and characterized by high proportion of green areas (as much as 63% according to Luniak et al.^50^). These are: numerous parks, squares, residential and roadside vegetation, cemeteries, botanical and zoological gardens, allotment gardens, home gardens, old city fortifications, open areas and agricultural land (pastures, meadows, orchards). Moreover, natural greenery like riparian forest along the Vistula and smaller watercourses, nature reserves and forests are important green areas. Most of parks and squares are located in the city centre (Fig. 1). Forests account for about 15% of the city area and are located mainly in the outskirts, and in the eastern and south-eastern part of the city (Fig. 1). It may be assumed that the connectivity between green areas on each side of the river is relatively maintained, and that the system of protected areas in Warsaw provides a safeguard to populations of most, especially small mammal species^51^.

### Research methods

The study included three stages: (1) assessment of the fox distribution in Warsaw, (2) describing the process of settlement in the selected green areas and (3) temporal and spatial patterns of incidents with red foxes.

### Data collection

#### Assessment of the red fox distribution in Warsaw

**Figure 2:**
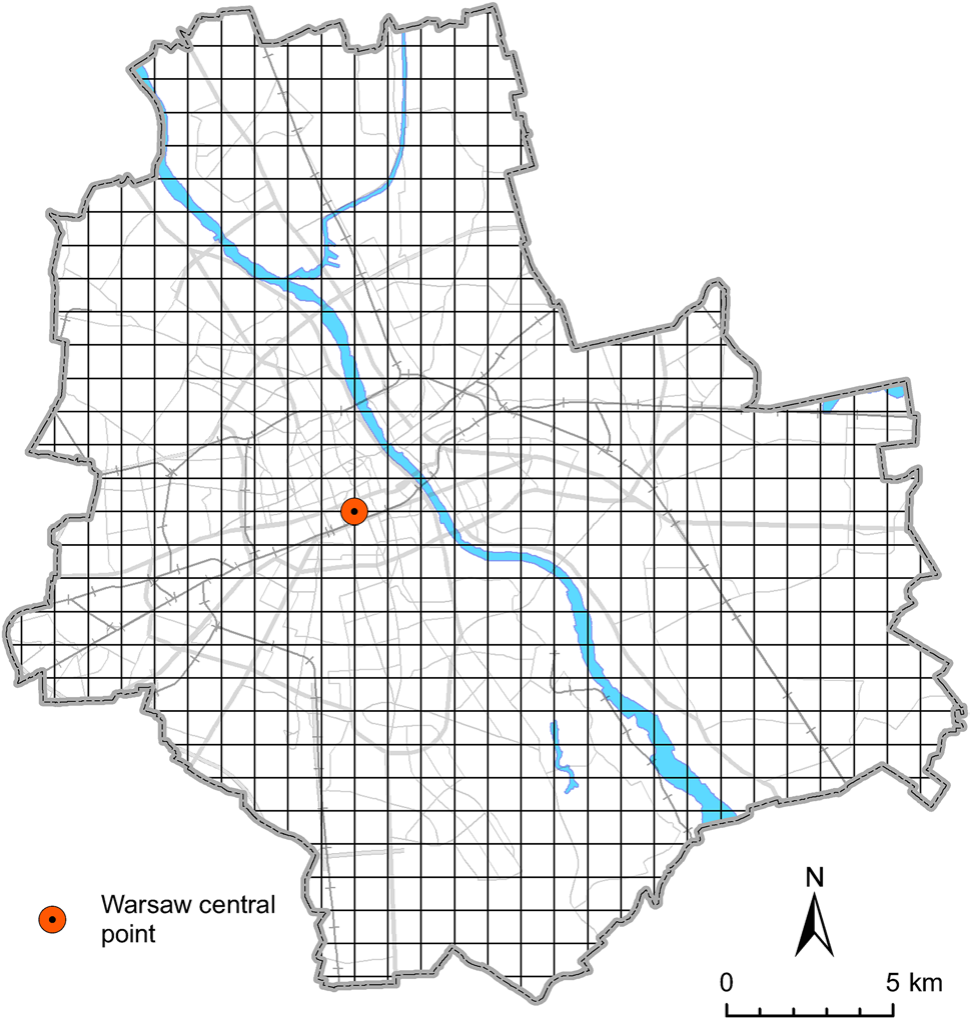
The grid of squares searched for presence signs to assess red fox distribution in Warsaw in 2005–2012. The Palace of Culture and Science, assumed as the most central point of the city is marked.

To assess the occurrence of the red fox in Warsaw, the city area was divided into grid of 593 squares (Fig. 2) with the resolution 1 × 1 km, 152 squares were located at the borders of Warsaw (with part being outside the city not included in the study). Each square was checked for the presence of the red fox. First, all previously collected direct sightings of red fox and known fox dens dating back to 2005 were assigned to a square. Next, all remaining squares were searched between 2011 and 2012 for signs of fox presence. Most of the data was collected during winter 2011–2012, when long-lasting snow cover allowed for efficient (snow tracking) survey. Additionally, occupied and non-occupied burrows, presence of adults or juveniles, or killed animals (mostly victims of collisions), and, in some cases, tracks on the mud/sand or scats were recorded. When some of the above was found, the square was marked as ‘fox present’ and no further checks were done in this particular square. When no signs were found, we repeated the survey or searched the square more thoroughly.

#### Process of the settlement of the red fox in the selected green areas

To estimate the progress in the settlement of selected green spaces in the city, 29 various green areas (4 to 139 ha) located in different distance from the city centre were selected. They were attributed to four different habitat types: cemeteries (n = 6), forests (n = 5), parks (n = 11) and riparian habitats in the Vistula river valley (n = 7). The green areas were easily identified as separate units, and they remained relatively stable in terms of boundaries and land use since the first compared period: the 1970s. The research was done in three periods: (1) 1976–1978 (Goszczyński J., unpbl. data), (2) 2004–2012 and (3) 2016–2019. In the first period, in all selected areas snow-tracking on transect routes (i.e. line transects) was done to confirm the species presence. In the next two periods, presence of fox was confirmed either on the basis of snow tracking on the transect routes or on the basis of presence of dens or breeding records within the green area. The total lengths of the line transects were: 113.9 km (on average 3.9 km in one study area, min = 0.15, max = 23.5 km), 61.0 km (2.8 km, min = 0.5, max = 9.1 m), 93.4 km (5.5 km, min = 0.8, max = 21.2 km) in the three periods, respectively.

#### Spatial and temporal patterns of incidents involving the red fox

As the third source of data, we used reports on red fox presence in the city (1998–2015), recorded by the Municipal Forests—Warsaw. The data included any kind of incident involving red fox: sightings (reported by city inhabitants), trapping attempts, culling or euthanasia, traffic collisions involving the species, observation of burrows or litters, individuals found dead or delivered to the rehabilitation centre governed by the Municipal Forests—Warsaw. For each incident, time and date, approximate or exact location (see Supplementary Fig. S1) and condition of the animal (alive, wounded, dead) were recorded.

### Statistical and spatial analysis

#### Process of the settlement of the red fox in the selected green areas

The χ^2^ test for goodness of fit was used to test the null hypothesis that the observed counts of objects (green areas) of each habitat type, colonised by foxes followed the expected counts, which were proportional to the total number of objects belonging to a given habitat category. In the case the null hypothesis had been falsified the post-hoc analysis was performed to determine which counts differed from their theoretical proportions. The confidence intervals for multinomial proportions were calculated using method proposed by Sision and Glaz^52^ with the function MultinomCI from package DescTools^53^.

#### Spatial and temporal patterns of incidents involving the red fox

To analyse temporal patterns of incidents with foxes two statistical models were built. The first one described annual and year to year variation (1998–2015) in the number of incidents with the red fox. The second one described the hourly variation in the number of incidents. We checked the assumption that incorporation of nonlinear effects to the model improved its performance by comparison of two models both fitted with gam function from library mgcv. The first had only linear terms and the second had nonlinear smoothers. To compare explanatory power of both models we used the anova function. The methods described above, confirmed with the significance level at least 0.05, that models containing non-linear terms performed better in the case of both models. We tested for concurvity (which is counterpart for collinearity for gam models) in the first gam model (containing more than one independent variable) with function concurvity() from library mgcv. We also checked for collinearity in each logistic regression model for spatial data with the use of function check_collinearity from library performance.

To build the first temporal model each recorded incident with fox was coded for ‘day of year’ (ordinal day as number ranging from 1 to 366) and ‘time of week’ (variable week with only two levels weekday and weekend). Based on these attributes the number of collisions per day was calculated for each day in the analysed period (6574 estimates). To account for a possible trend in the number of fox incidents in the analysed period the additional variable ‘time of data collection’ was introduced. This was based on a unique number representing a calendar day in R software environment. The variable was scaled by 1000 to values in a range from 10,227 to 16,800 representing all days in the analysed period. The general additive model (GAM) with a Poisson error distribution and log link was used to determine how the number of incidents changed over time during 1998–2015. These kind of models use splines, that allow for determination of nonlinear fit between dependent and independent variables in a more elastic manner then parametric nonlinear functions usually implemented in generalized models. The ‘number of incidents per day’ was used as a response variable. The ‘day of year’ and described above variables: ‘time of data collection’ and ‘time of week’ were used as explanatory variables. In the GAM model an explanatory variable ‘day of year’ was fitted with cyclic penalized cubic regression splines whose ends match (i.e., number of incidents in day 1 and day 366 was assumed to be the same; Wood^54^). The variable ‘time of data collection’ was fitted in a model with ordinary regression splines. For both mentioned variables the upper limit for the effective degrees of freedom of the spline was set at 10 to control the complexity of the fit. The variable ‘time of week’ was used as fixed categorical variable.

The second temporal model was built to estimate the daily variability of incidents with foxes. The dependent variable was calculated as a number of incidents per minute of a day, e.g. all cases of fox incidents which happened between 0:00 and 0:01 in the whole period of study (between 1998 and 2015) were pooled and assigned to the first minute of a day. This gave variable containing 1440 min estimates. The only independent variable used in this model was the ‘minute of day’ ranging from 1 to 1440. As in previous model the explanatory variable was fitted with cyclic penalized cubic regression splines whose ends match and the effective degrees of freedom of the spline was set at 10. Both GAM models were parametrized using the mgcv package^55^.

Among all incidents with red foxes, only those for which the exact location was known (i.e. the street number or other details that allowed to locate the place with at least 50 m accuracy were given) were taken for spatial analysis. In order to analyse habitat characteristics for red fox incidents locations, the following data were involved: Urban Atlas (UA) published in 2018 and derived by EU Copernicus Land Monitoring Service^56^, which presented the biophysical characteristic of Earth, the polygon data for the Vistula river and the central point of Warsaw. The UA layer was generalized into seven classes of landcover (continuous urban fabric, discontinuous urban fabric, industrial areas, parks, agricultural areas, forests, wetlands; see Supplementary Table S1). Around all fox incident locations the circle polygons with the radius 100, 250, 500 and 1500 m were generated by buffer function. Next UA layers were intersected with each buffer independently and the UA classes percentages for each radius were calculated. The planar distances between fox incident location and aforementioned point layers were done by the ArcGIS near function. The same procedure was repeated for the 1000 randomly generated points within the border of Warsaw. To analyse spatial characteristics of incidents four logistic regression models were built. The dependent variable was a binary vector of length 1263, taking values equal 1 for all 263 incidents with foxes for which precise geographic location was available and 0 for additional 1000 locations, randomly distributed within the border of Warsaw. The distance to the city centre (i.e. the most central point of the city, Fig. 1), the distance to the Vistula River (an ecological corridor within the city) and the percentage share of seven habitats that were subject to different level of anthropopression (Supplementary Table S1) in the buffer zones (100, 250, 500, 1500 m) were taken as independent variables in each model. In each spatial model the characteristic of surrounding habitats were calculated based on different radius 100, 250, 500, 1500 m. Variables that were not important statistically were removed from fitted models. The comparison of model performance based on R^2^ could be used to judge which environmental variables and in a given spatial scale were the most important to predict incidents with foxes.

All statistical calculations were performed in R^57^. All spatial analyses were performed in ArcGIS package version 10.8.1^58^.

## Results

### Assessment of the red fox distribution in Warsaw

Signs of red fox presence were found in the whole city, i.e. the fox presence was confirmed in each of 593 1 × 1 km^2^ of the grid checked between 2005 and 2012.

### Process of the settlement of the red fox in the selected green areas

**Figure 3:**
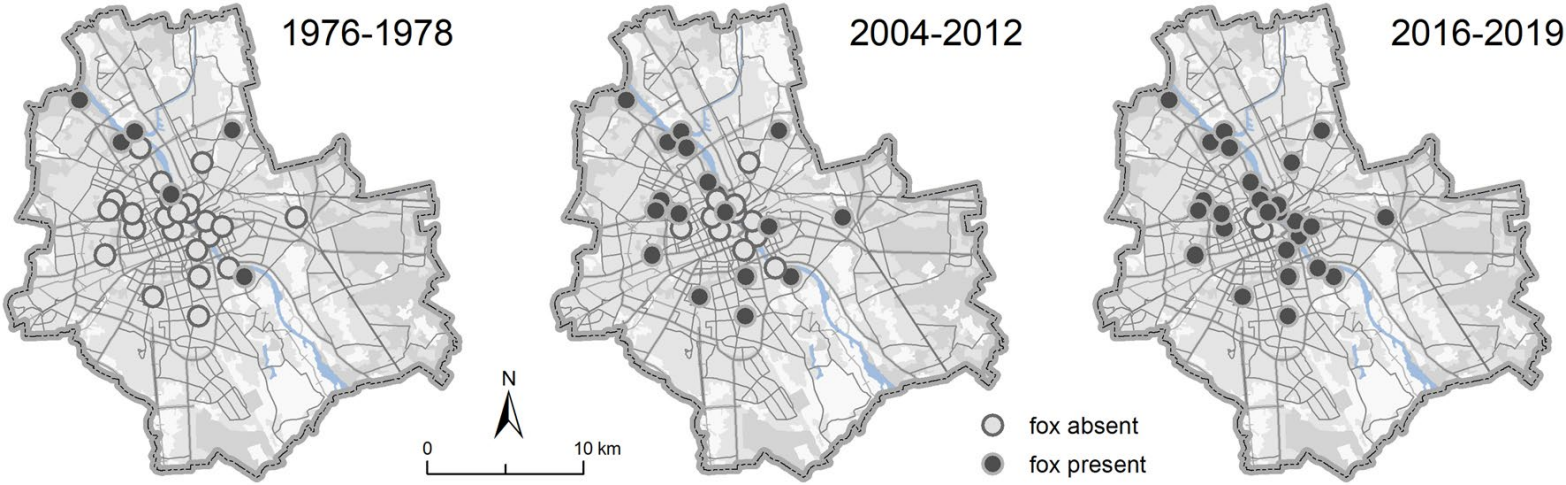
Presence of the red fox as confirmed by snow tracking in 29 randomly selected green areas in Warsaw in the three study periods (data for 1976–1978 after Goszczyński J., unpbl.).

**Figure 4:**
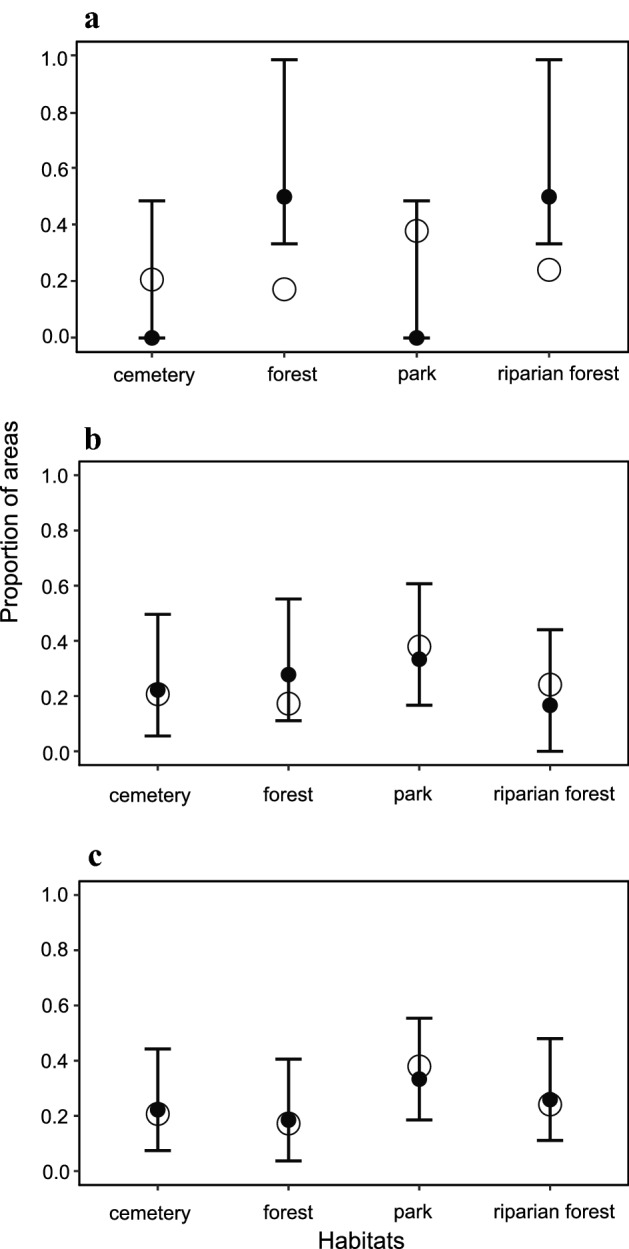
Proportion of green areas occupied by the red fox as confirmed by snow tracking, attributed to four habitat types in Warsaw, in subsequent study periods: (**a**) 1976–1978 (Goszczyński J., unpbl. data), (**b**) 2004–2012 and (**c**) 2016–2019. A full circle indicates actual proportion and an empty circle indicates expected proportion of colonized areas attributed to a certain habitat type, bars depict lower and upper bound of the confidence interval.

In 1976 to 1978, only six (21%) of 29 selected green areas were colonized by foxes. This increased to 18 (62%) in 2004–2012 and 27 (93%) in 2016–2019 (Fig. 3). Areas covered by forests and riparian habitats (Supplementary Table S2) were occupied in the first study period (1976–1978) more frequently (test for goodness of fit, *p* = 0.031). For the other two periods no significant differences in the selection of habitats were found (*p* = 0.638 and *p* = 0.971, respectively) (Fig. 4).

### Temporal pattern of incidents involving the red fox

In the years 1998–2015, 608 incidents involving foxes were reported; 52% of them concerned alive individuals (mostly fox sightings or trapping attempts), in 5% injured foxes were reported. In 47% of cases foxes were found dead (mostly due to traffic collisions). The number of reported incidents per year increased gradually from just a few cases in 1998 and 1999 to over 80 in 2015. The number of city districts where incidents were reported also increased gradually from four and two in 1998 and 1999, respectively to 16 (out of total 18) in 2015. The highest number of reported incidents per day was three.

**Figure 5:**
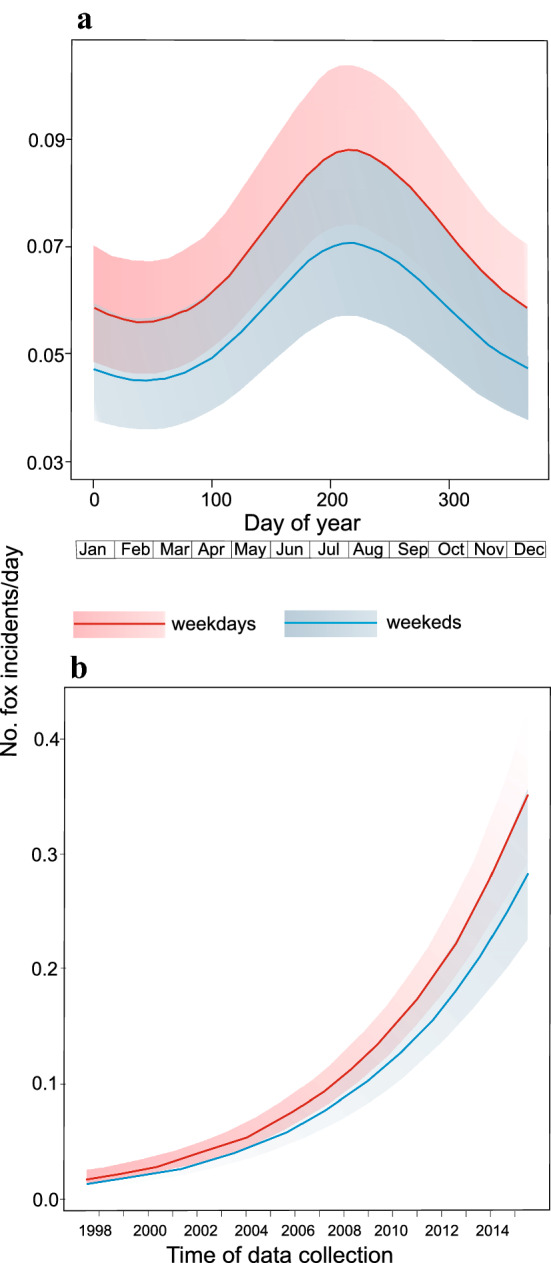
Probability of the red fox incident (**a**) during a year and (**b**) in the whole research period with comparison between weekdays and weekends as based on the reports on fox incidents in the city area (1998–2015), delivered by the Municipal Forests—Warsaw.

All three time scale predictors (time of week, day of year, time of data collection) included in the GAM significantly influenced the probability of reporting a fox incident (Table 1). The higher probability was from June to October, and most of the reports (58.6%, N = 314) in this period concerned fox sightings or trapping attempts. In the remaining months, this probability was lower, and the majority of incidents involved dead animals (54.3%, N = 289). Overall, the probability of reporting the red fox incidents in Warsaw increased over study course (Fig. 5), and was significantly affected (*p* = 0.022) by time of week, being higher on working days than weekends (Fig. 5).

**Table 1:** Results of generalized additive models explaining probability of the red fox incidents in Warsaw.

Predictors	Estimate	SE	z or χ^2^	*p*
**Model 1 (R**^**2**^_**Nagelkerke**_** = 15.9%)**				
Intercept	− 2.69	0.06	− 44.10	< 0.001
Time of week	− 0.22	0.09	− 2.30	0.022
Day of year	edf = 2.21	–	18.39	< 0.001
Time of data collection	edf = 1.45	–	275.80	< 0.001
**Model 2 (R**^**2**^_**Nagelkerke**_** = 10.9%)**				
Intercept	− 1.31	0.12	− 11.21	< 0.001
Time of day	edf = 3.34	–	39.71	< 0.001

‘Model 1’ analyzing the effect of time of week (weekdays vs. weekends), day of year (1–366) and time of data collection (a day in the period 1998–2015), on the probability of an incident with the red fox in Warsaw and the second one, ‘model 2’ analyzing the impact of time of day (hour) on the probability of fox incident. Estimated number of degrees of freedom, standard error, z or *χ*^2^ statistics, *p* value and explanatory power of each model (R^2^_Nagelkerke_) are given.

**Figure 6:**
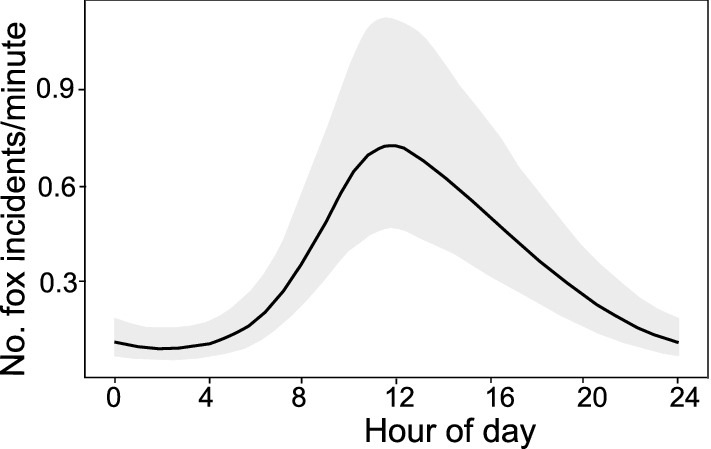
Modelled number of the red fox incidents per minute during the day as based on the reports on the red fox incidents in the city area (1998–2015), delivered by the Municipal Forests—Warsaw.

Time of day (*p* < 0.001) also influenced the probability of a red fox incident. The incidents were reported most often between 8 a.m. and 8 p.m. with a clear peak around noon. The biggest chance of incident report was between 10.00 a.m. to 02.00 p.m., and lowest between 08.00 p.m. to 07.00 a.m. (Fig. 6).

### Spatial patterns of incidents involving the red fox

**Figure 7:**
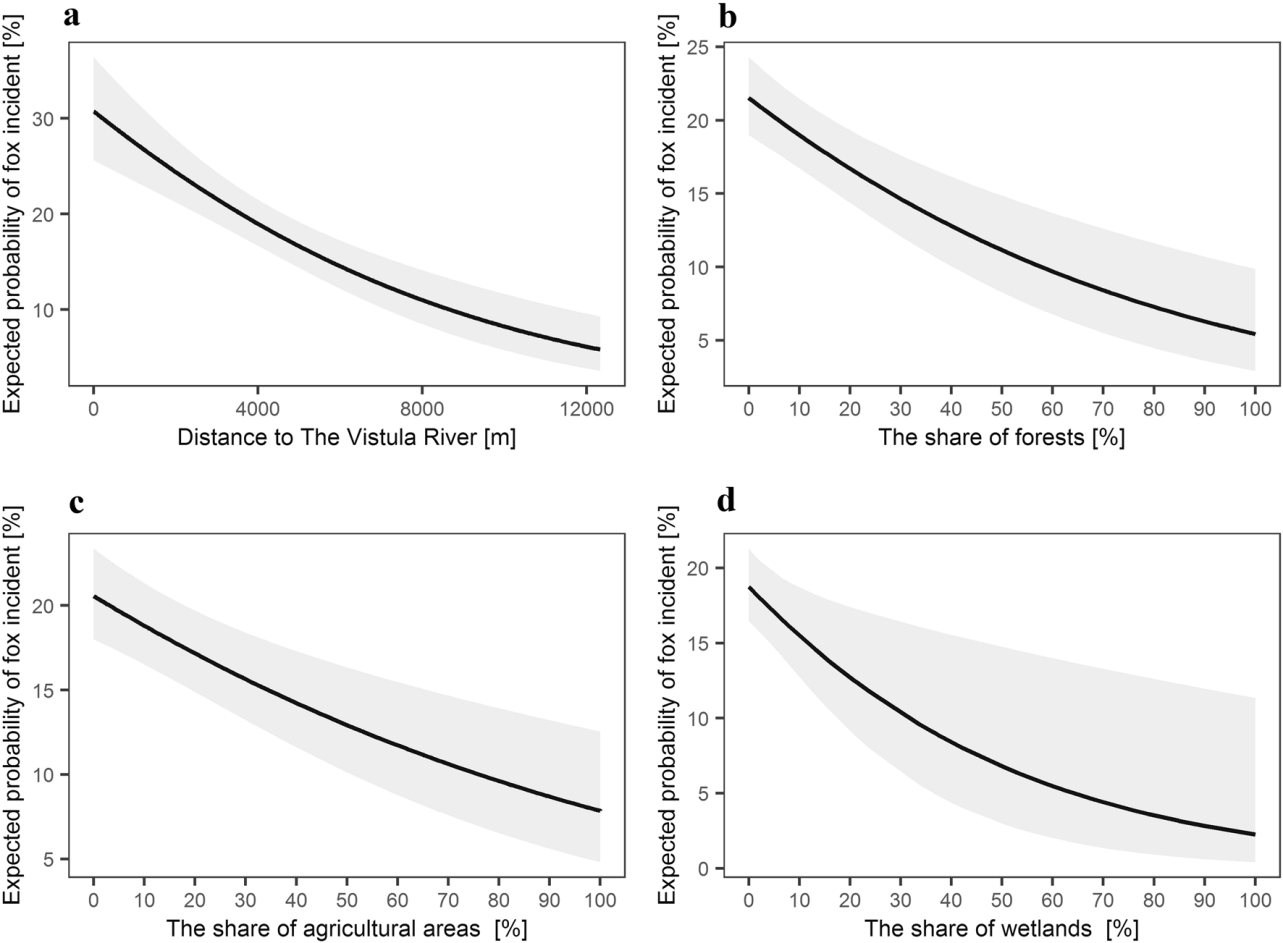
Expected probability of the red fox incidents as based on the reports (1998–2015), delivered by the Municipal Forests—Warsaw, in relation to distance to (**a**) the Vistula river (an ecological corridor), (**b**) share of forest, (**c**) share of agricultural areas, and (**d**) share of wetlands within 100 m radius around location of red fox incident. Explanatory variables that were significant in the logistic regression model (see Table 2) are shown.

**Figure 8:**
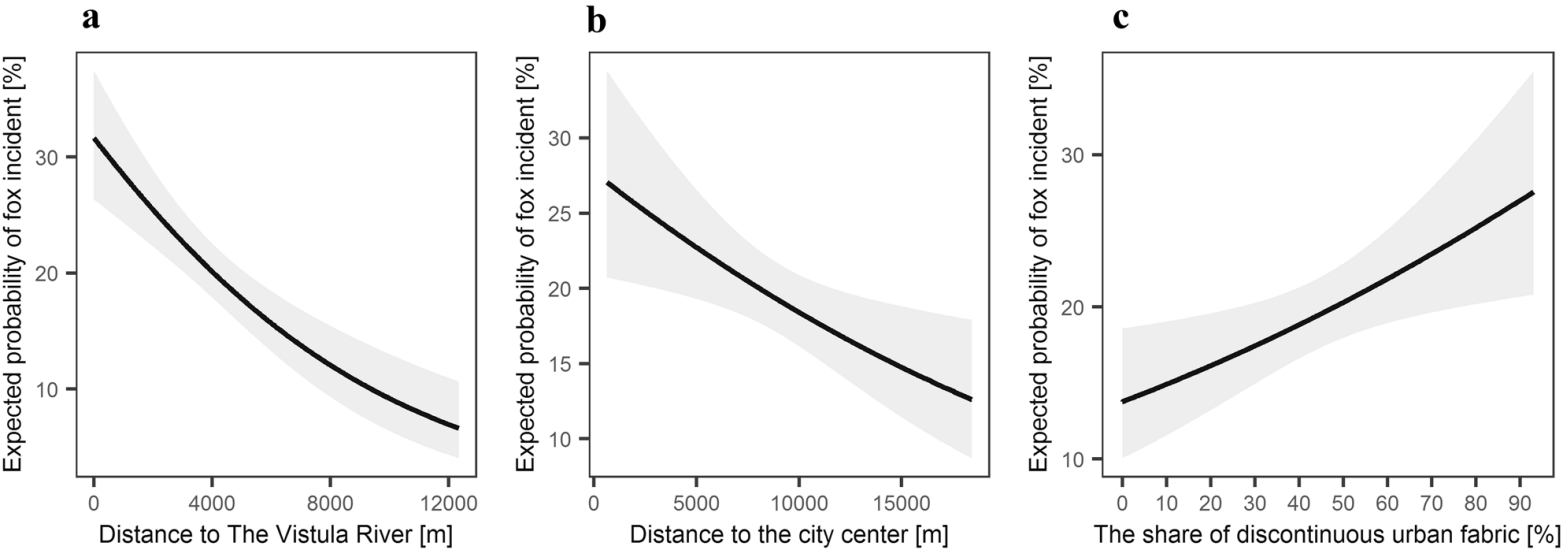
Probability of the red fox incident as based on the reports (1998–2015), delivered by the Municipal Forests—Warsaw, in relation to distance to (**a**) the Vistula river, (**b**) the city centre and (**c**) share of discontinuous urban fabric within 1500 m radius around location of red fox incident. Explanatory variables that were significant in the logistic regression model (see Table 2) are shown.

We recorded a general negative relationship with the proportion of forests, agricultural areas, wetlands and with an increasing distance to the Vistula River for the buffers with radius 100, 250 and 500 m (Table 2, Fig. 7). Within 1500 m radius buffer there was a negative relationship with an increasing distance to the Vistula River and the city centre, and a positive with the share of discontinuous urban fabric (Table 2, Fig. 8). No collinearity was found between the variables.

**Table 2:** Influence of habitat characteristics on the probability if the red fox incident in Warsaw.

Predictors	Estimate	SE	Z	*p*
**100 m radius (R**^**2**^_**Tjur**_** = 0.069; AUC = 0.684)**				
Intercept	− 0.38845	0.12804	− 3.03368	0.002
Distance to the Vistula river	− 0.00016	0.00003	− 5.69064	< 0.001
Share of forests	− 0.00005	0.00001	− 4.58908	< 0.001
Share of agricultural areas	− 0.00004	0.00001	− 4.00039	< 0.001
Share of wetlands	− 0.00007	0.00003	− 2.62905	0.009
**250 m radius (R**^**2**^_**Tjur**_** = 0.065; AUC = 0.678)**				
Intercept	− 0.34782	0.13128	− 2.64937	0.008
Distance to the Vistula River	− 0.00017	0.00003	− 5.80572	< 0.001
Share of forests	− 0.00001	0.00000	− 4.42650	< 0.001
Share of agricultural areas	− 0.00001	0.00000	− 3.55513	< 0.001
Share of wetlands	− 0.00001	0.00001	− 2.79133	0.005
**500 m radius (R**^**2**^_**Tjur**_** = 0.060; AUC = 0.672)**				
Intercept	− 0.29848	0.13850	− 2.15513	0.031
Distance to the Vistula river	− 0.00017	0.00003	− 5.74674	< 0.001
Share of forests	− 0.00000	0.00000	− 4.19826	< 0.001
Share of agricultural areas	− 0.00000	0.00000	− 3.32188	0.001
SHARE of wetlands	− 0.00000	0.00000	− 2.71936	0.007
**1500 m radius (R**^**2**^_**Tjur**_** = 0.045; AUC = 0.648)**				
Intercept	− 0.69091	0.25402	− 2.71995	0.007
Distance to the Vistula river	− 0.00015	0.00003	− 5.16528	< 0.001
Distance to the city centre	− 0.00005	0.00002	− 2.63165	0.008
Share of discontinuous urban fabric	0.00000	0.00000	2.56185	0.010

Results of the logistic regression models for 100, 250, 500 and 1500 buffer radius around location of red fox incident in Warsaw in 1998–2015 has been presented. The explanatory ability of models (R^2^_Tjur_ and AUC) is given.

## Discussion

The red fox is one of the mammal species that successfully colonized many cities around the world^37^. Its presence was confirmed in Warsaw more than a few decades ago. Nevertheless, at those times the red fox was assumed to inhabit mainly fairly natural habitats (i.e. forests) and remote city areas. The aim of our study was to describe the process of Warsaw colonization by the red fox in recent decades. A concept of the process of city colonization assumes division into three phases: (1) arrival, (2) adjustment and (3) spread^4^. In this paper we focused on the spread of the species in the city. The red fox arrival in Warsaw is hard to estimate, yet first data on the red fox in Warsaw dates back to 1890s^59^. Low population density and few sightings in the 1970s [Goszczyński J., unpublished] may suggest that proper spread of the red fox in Warsaw started no sooner than in the last decades. As we showed on the basis of presence-absence data through the grid of squares, red foxes had already started to penetrate the whole area of Warsaw. In the past decades (i.e. in the 1970s), it inhabited mostly green spaces of more natural character (i.e. forests), while in the XXI century parks and cemeteries were occupied as often.

The red fox colonizes urban areas because of its extraordinary adaptability to settle in new habitats. Animals may be also pushed into the city as a result of overpopulation in non-urban areas^36^. Previous molecular studies on colonization of urban habitats by the red fox showed that the process starts usually from a few locations near the city borders and spreads gradually into its central parts^60,61^. The red fox movements through the city can be affected by human-made infrastructures^62^, like railways^63^, roads^62,64,65^. Densely built-up areas are usually avoided by foxes^14,62^, while specific migration corridors like roadside or railway vegetation^62,66^ will facilitate the process. Indeed, in Warsaw the red fox inhabited at first the largest green areas located mainly in the outer city districts or located in the Vistula riparian forests, a natural ecological corridor for mammals^48^. At the next stage, a number of green spaces occupied by the red fox increased. City habitats are generally readily accessible by foxes (see Douglas and Sadler^67^), therefore after several decades of the colonization process in Warsaw, only a small percentage of green areas under study was not used by foxes. They were highly isolated, small urban parks, often visited by people, with limited availability of potential den sites. They were also located close to or in the strict city centre, areas usually avoided by foxes^13,68,69^.

The other part of our research was based on data derived from the Municipal Forests in Warsaw, the administration unit dealing with and collecting data on incidents with wildlife in Warsaw. This included mostly any incidental observational data by city inhabitants or cases of traffic collisions. Such data was already used by other researchers to study encounters between carnivores and humans or urban population trends^15,21,28,31,70^ and here we wanted to evaluate its usefulness in future studies. We showed that the probability of reporting on incident involving red foxes was dependent on different temporal variables. First, most of the incidents were noticed during a peak of a day (between 10.00 am and 02.00 pm), which is atypical fox activity (regardless of habitat type) compared with most other studies^18,28,31,39,71–73^. However, it was shown that the daily activity of urban populations of the red fox differed. In Great Britain, in a typical urban population, foxes were observed mostly during the day^40^, while in Melbourne (Australia), red fox sightings at this time were very rare. This was explained by the initial stage of population development^21^. Therefore, we assume that the probability of reporting incidents with the red fox in our case was dependent on human rather than red fox activity. The number of incidents involving red foxes increased beginning from 07.00 a.m. and clearly declined after 08.00 p.m., what corresponds with highest number of people staying outdoor^74^. In Finland, Kauhala et al.^31^ came to the same conclusion with even clearer peaks of observations at a few specific hours during the day. Next, we showed that the number of reported red fox incidents on weekdays was higher when compared to weekends. This also may be attributed to higher weekday outdoor activity of city inhabitants and higher traffic volume, thus increased probability of the red fox sighting or vehicle collision. Finally, we showed that the peak of the red fox incidents was reported in summer and early autumn (June–October) while being lower in the remaining months (November–April). Similar distribution of observations during the year was in Turku in Finland^31^, but different in Estonian cities^28^. Again, we may assume that in our case, this can be explained by both circannual activity of the red fox but also human outdoor activity. February to April is the birth time of foxes, with spatial activity (especially of vixens) limited mainly to the area of breeding dens^39,75–77^, which is in line with recorded low number of red fox incidents at that time. However, December to February is the mating season of red foxes when increased activity of males is observed^77^, so a higher number of incidents could be expected. Here, low number of the red fox incidents was probably the result of low human activity (as connected to unfavourable winter weather conditions) rather than fox activity. Similarly, the peak of incidents between June and October can be related to high red fox activity (especially increasing range of juveniles^78^) but also higher human outdoor activity in summer and early autumn months.

This relation between the number of the red fox incidents and human outdoor activity is also visible when spatial patterns are analysed. The number of the red fox incidents decreased with the share of forests, meadows, wetlands (which are natural red fox habitats^79^) but increased with the share of sparsely built-up areas. It was also higher close to the city centre and to the Vistula River. Foxes inhabiting residential gardens and low-density housing areas were observed in other cities^14,80,81^, yet in Warsaw dens of red foxes are hardly ever recorded in such surrounding, they are mostly located in forests (Jackowiak M., unpbl.). At the same time, in the city centre and in areas with low-density housing the outdoor activity and presence of people is high^82^, which may favour more frequent fox observations. The Vistula River in turn, goes through the whole of Warsaw (including its city centre), serving as an ecological corridor but also a popular recreational area, so wildlife-human encounters here are likely. Similar results were obtained in Turku^31^ and in Vienna^71^.

The eagerness to report the fact of observing a wild animal, depends on observer’s motivation^26^, and in our study may be explained by people concerned about the presence of carnivorous mammals (in this case the red fox) in cities. As revealed in a questionnaire study in Germany, the majority of inhabitants were not afraid of foxes and were generally pleased with fox presence, yet had their concerns about potential zoonotic diseases^30^. The red fox is one of the most important vectors of zoonoses and their presence in cities may affect the spread of epidemic risks^31,83–85^. In Poland and in Warsaw, rabies, as the most dangerous fox-transmitted disease, is still present in fox population^86^ but also frequent cases of mange occur (Jackowiak M., unpbl.). Due to this red fox encounters arise usually anxiety in city inhabitants who, in such situations, may willingly seek help from wildlife services.

Although, human activity influences the number of the red fox observations^43,71^, fox sightings were used to estimate population abundance and how it changed (e.g.^20,36,42,87^). In our study, the probability of a red fox incident rose over time, which points to a population increase. Yet, it should be kept in mind that during our study, access to the Internet (thus information on urban wildlife), and mobile phones increased rapidly, which possibly also boosted the number of the red fox incidents reported.

In our study we showed the process of red fox settlement in Warsaw, the capital city of Poland. At first, the red fox colonised mainly green areas of more natural character and placed further from the city centre, gradually expanding throughout the city. The red fox requires three factors that are crucial for urban population existence—access to daytime resting sites, convenient breeding sites and food availability^88^. Wide distribution of the red fox in Warsaw proves that these conditions are fulfilled and the abundance of the red fox population will probably further increase. For decades, red fox was heavily hunted (and poached) as pest (preying on small game and domestic animals, mostly poultry) but also for its valuable fur^79^ so red foxes avoided people (e.g. they had their dens almost exclusively in forests^89^). This has changed in the last (approximately) twenty years, i.e. red fox abundance increased thanks to anti-rabies vaccinations^90^. This increase in population abundance and, assumingly, lowering fear of human will lead to higher tolerance of red foxes to closer contacts with people. With the red fox settlements in more human-transformed habitats within city boundaries, red fox-human encounters (and possibly also conflicts) will also increase. This will affect epidemiological risks. Thus, the urban red fox monitoring remains essential for the risk assessment and proper population management. Nevertheless, as we shown in this study, incidental red fox sightings by city inhabitants should be treated with caution. Being highly influenced by patterns of human outdoor activity and people attitudes towards wildlife (driven also by health concerns) they only partially reflect real population abundance trends as well as temporal and spatial activity patterns of this medium-sized carnivore.

## Supplementary Information

Supplementary Information.
