# Supplementary material for: Colonization of Warsaw by the red fox Vulpes vulpes in the years 1976–2019
Source: Sci Rep. 2021 Jul 6;11:13931. doi: 10.1038/s41598-021-92844-2 (PMC8260808; doi:10.1038/s41598-021-92844-2)
Supplement: Supplementary file 1 — Supplementary Information. [file 41598_2021_92844_MOESM1_ESM.pdf]

SUPPLEMENTARY INFORMATION FILE

Colonization of Warsaw by the red fox *Vulpes vulpes* in the years 1976-2019

Mateusz Jackowiak, Jakub Gryz, Karolina Jasińska, Michał Brach, Leszek Bolibok, Piotr Kowal, Dagny Krauze-Gryz

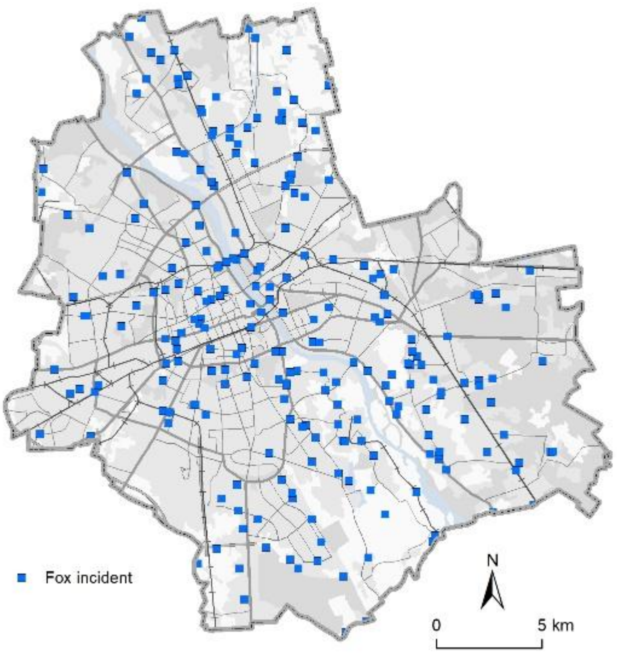

Fig S1. The location of incidents involving the red fox in Warsaw in the years 1998-2015 as based on the reports (1998-2015), delivered by the Municipal Forests – Warsaw.

Table S1. Classes of generalized Urban Atlas (UA) layer [56], used in spatial analysis of the red fox incidents in Warsaw

Class number	Class name	Description
1	Continuous urban	Continuous urban fabric: buildings, roads and sealed areas cover most of the area; non-linear areas of vegetation and bare soil are exceptional. Average degree of soil sealing >80%.
2	Discontinuous urban	Discontinuous urban fabric: build-up areas; a larger fraction (as opposed to continuous urban fabric) of non-sealed and/or vegetated surfaces (gardens, parks, planted areas and non-surfaced public areas) is included. Average degree of imperviousness/soil sealing is between 0 to 80%.
3	Industrial	Industrial or commercial units, road and rail networks and associated land, port areas, airports, mineral extraction sites, dump sites, construction sites
4	Parks	Green urban areas, sport and leisure facilities
5	Agricultural areas	Arable land, non-irrigated arable land, permanently irrigated land, vineyards, permanent crops, fruit trees and berry plantations, heterogeneous agricultural areas
6	Forests	Broad-leaved forest, coniferous forest, mixed forest, natural grasslands
7	Wetlands	Inland wetlands, inland marshes, water courses, water bodies, estuaries

Table S2. Characteristics of green areas, used to monitor the progress of Warsaw colonization by the red fox. 29 randomly selected areas has been chosen to monitor progress of city colonization by the red fox in subsequent years: 1976-1978 (Goszczyński J., unpbl. data), 2004-2012 and 2016-2019. The table shows the name of the area, type of a habitat, surface, distance to the city centre and the presence (+)/absence (-) of red fox based on winter tracking.

Area	habitat type	surface [ha]	distance to the city centre*[km]	fox presence		
				1976-1978	2004-2012	2016-2019
Bródno Cemetery	cemetery	113	5.71	-	-	+
Evangelical-Augsburg Cemetery (Wola)	cemetery	7	2.62	-	-	+
Powązki Cemetery	cemetery	43	3.30	-	+	+
Orthodox Cemetery (Wola)	cemetery	13	2.79	-	+	+
Powązki Military Cemetery	cemetery	24	4.66	-	+	+
Soviet Military Cemetery	cemetery	19	3.47	-	+	+
Bielany Forest	forest	130	7.57	+	+	+
Bródno Forest	forest	134	8.27	+	+	+
Nowa Warszawa Forest	forest	139	10.71	+	+	+
Koło Forest	forest	44	4.45	-	+	+
The Vistula River valley:						
vicinity of Poniatowski Bridge (left bank)	riparian forest	-	2.63	-	-	+
Praga Harbour and vicinity of Wybrzeże Szczecińskie Street	riparian forest	-	2.49	-	-	+
vicinity of Śląsko-Dąbrowski	riparian	-	2.41	-	-	+

Bridge (left bank)	forest					
Żerań Harbour and Żerań Channel	riparian forest	-	6.94	+	+	+
vicinity of Bartycka Street	riparian forest	-	3.95	+	+	+
vicinity of the water intake	riparian forest	-	3.69	-	-	+
Gdański Bridge (left bank)	riparian forest	-	3.30	+	+	+
Saxon Garden	park	16	1.00	-	-	-
Arkadia Park	park	11	4.87	-	+	+
Fos Park	park	18	3.92	-	+	+
Marshal Rydz-Śmigły Park	park	53	1.65	-	-	+
Kępa Potocka Park	park	18	5.34	-	+	+
Krasiński Garden	park	12	1.89	-	-	-
Royal Łazienki Park	park	76	2.68	-	+	+
Praga Park (and ZOO)	park	58	2.84	-	-	+
Skaryszew Park	park	58	3.51	-	+	+
Traugutt Park	park	10	2.90	-	-	+
Olszynka Grochowska Nature Reserve	forest	57	8.11	-	+	+
Powstańcy Styczniowi Square	park	5	8.11	-	+	+

* measured to the Palace of Culture and Science, assumed as the most central point of the city
